# Rapid and sensitive detection of *Chlamydia trachomatis* sexually transmitted infections in resource-constrained settings in Thailand at the point-of-care

**DOI:** 10.1371/journal.pntd.0006900

**Published:** 2018-12-20

**Authors:** Naraporn Somboonna, Ilada Choopara, Narong Arunrut, Kanchapan Sukhonpan, Jarun Sayasathid, Deborah Dean, Wansika Kiatpathomchai

**Affiliations:** 1 Department of Microbiology, Faculty of Science, Chulalongkorn University, Bangkok, Thailand; 2 Program in Biotechnology, Faculty of Science, Chulalongkorn University, Bangkok, Thailand; 3 Bioengineering and Sensing Technology Laboratory, National Center for Genetic Engineering and Biotechnology, Khlong Nueng, Khlong Luang, Pathum Thani, Thailand; 4 Department of Obstetrics and Gynecology, Buddhachinaraj Phitsanulok Hospital, Phitsanulok, Thailand; 5 Cardiac Center, Naresuan University Hospital, Phitsanulok, Thailand; 6 Center for Immunobiology and Vaccine Development, UCSF Benioff Children's Hospital Oakland Research Institute, Oakland, California, United States of America; 7 Department of Medicine, University of California, San Francisco, San Francisco, California, United States of America; 8 Joint Graduate Program in Bioengineering, University of California, Berkeley, Berkeley, California, United States of America; University of Tennessee, UNITED STATES

## Introduction

*Chlamydia trachomatis* is the leading cause of sexually transmitted diseases (STDs) in females and males in both developed and developing countries, with more than 110 million cases annually. *C*. *trachomatis* resists antibiotic treatment and is a cofactor in HIV transmission and human cervical cancer [1]. Infection is often asymptomatic, causing the epidemiology to be underestimated. Therefore, we have developed a rapid, inexpensive, easy-to-interpret, sensitive and specific point-of-care (POC) *C*. *trachomatis* detection system, using loop-mediated isothermal amplification (LAMP) for target *C*. *trachomatis* DNA amplification, followed by gold nanoparticle probe (AuNP) for colorimetric *C*. *trachomatis* specific readout. The assay was evaluated using clinical samples and compared with polymerase chain reaction (PCR) of the same target gene, which is an outer membrane protein A (*ompA*) gene and a respected standard for *C*. *trachomatis* detection [1,2].

For nucleic acid amplification tests, recently LAMP has presented an attractive alternative to standard methods like PCR due to its low price, ease of use, rapid results, and lack of requirement for an expensive thermal cycler and specialized kits for DNA extraction and purification. A simple 5-minute boil for crude DNA lysis is sufficient for the LAMP reaction because the *Bacillus stearothermophilus* (*Bst*) DNA polymerase in LAMP has fewer inhibitor problems than *Thermus aquaticus* (*Taq*) DNA polymerase in PCR [3]. LAMP amplifies the target at a single temperature with high sensitivity (from 10 to 100 genome copies), and the product can be visualized as a white magnesium pyrophosphate precipitate. However, nebulous precipitate has the potential to cause misreading [3,4], so analysis by agarose gel-electrophoresis (GE), as with PCR, is standard. The positive LAMP reaction appears as multiple bands because several primers amplify amplicons of different sizes that are intercalated. Nevertheless, GE requires an electrophoresis apparatus, time, and often ethidium bromide exposure. In the current study, crude DNA lysis with combined LAMP-AuNP for POC *C*. *trachomatis* detection has been developed, and the specificity and limit of detection were validated by several positive and negative *C*. *trachomatis* genomes, as well as the possibility for POC diagnostic based on a statistical number of clinical endocervical swab sample tests (see Box 1).

**Box 1: Advantages and disadvantages of *C*. *trachomatis* LAMP-AuNP.**

**Advantages**

- Sensitive to as low as 11.25 copies of target DNA
- Total assay time is <1 hour
- Evaluation in independent replicates in 130 clinical samples showed 96% sensitivity and 99% to 100% specificity

**Disadvantages**

- Cannot diagnose the severity of disease
- Reaction reagents are in liquid
- Color change observation may be difficult for low copy numbers of *Chlamydia trachomatis*

## Materials and methods

### Study design and clinical sample collections

The criteria for symptomatic *C*. *trachomatis* was obtained from the description by the Centers for Disease Control and Prevention (CDC), such as abnormal cervical or vaginal discharge, elevated number of white blood cells in vaginal secretion, and co-infection with *Neosseria gonorrhoeae* [1]. Endocervical swab samples were randomly selected from a prospective study cohort of STD prevalence in symptomatic and healthy (which may include nonsymptomatic patients) Thai women aged 15 to 54 years in Bangkok and nearby areas. The samples were collected by clinicians during 2011 and 2012 from qualified volunteers attending the Buddhachinaraj Phitsanulok Hospital, and in 2013 and 2014 from Bangrak and Chonburi offices of Disease Prevention and Control [2]. The sample size of 130 (96 symptomatic and 34 healthy) was computed based on a standard statistical formula (http://www.calculator.net/sample-size-calculator.html), given the prevalence rates of *C*. *trachomatis* as 22% for symptomatic STDs and 3% for healthy people [2], with a margin of error of 0.05, and a 90% confidence interval. The samples were stored in 3 mL standard *C*. *trachomatis* collection medium M4RT (Thermo Fisher Scientific, Kansas, USA) at −80°C.

### Ethics statement

The study was approved by the Institutional Review Board of Buddhachinaraj Phitsanulok Hospital (101/54) and the Ethics Committee for Research in Human Subjects of the Department of Disease Control, Bangkok (FWA00013622). All subjects were provided written informed consent.

### *C*. *trachomatis* detection by commercial DNA extraction kit and PCR-GE

One milliliter of each endocervical swab sample in M4RT was pelleted at ≥12,000×*g* for 30 minutes at 4°C; then the pellet was extracted to obtain purified bacterial nucleic acids in 100 μl elution, using High Pure PCR Template Preparation Kit (Roche Diagnostics, Indianapolis, IN), following the manufacturer's protocols. For PCR, the Ct.ompA.MVF3 and Ct.ompA.220DR primers, as previously established, were used [5,6]. The PCR reaction contained 100 ng of DNA template (1–3 μl elution), 0.3 μM of forward and reverse primers, and 12.5 μl EmeraldAmp GT PCR Master Mix (TakaRa Bio, Shiga, Japan). The thermocycling profile was 94°C for 3 minutes—35 cycles of 94°C for 0.45 minutes, 45°C for 1 minute, and 72°C for 2 minutes—followed by 72°C for 10 minutes; 1.5% GE was performed to visualize the product.

### LAMP primers and design

A set of six primers were designed for LAMP to target eight distinct regions on the *C*. *trachomatis ompA* gene. First, *ompA* sequences from all 19 *C*. *trachomatis* serological variants (serovars: A to K, Ba, Da, Ia, Ja, L_1_, L_2_, L_2_a, L_2_b, L_2_c, and L_3_) were aligned using ClustalW (http://www.megasoftware.net/), and LAMP degenerate primers including loop primers LF and LB were designed using Primer Explorer V4 (http://primerexplorer.jp/elamp4.0.0/index.html) and manual design (Table 1). Two inner loop primers were included to speed up the reaction time. Primer specificity was verified by BLASTN. The primers were synthesized by Macrogen, Seoul, Korea.

**Table 1:** Primer and probe sequences of LAMP-AuNP for *C*. *trachomatis* detection.

Primer and Probe Name	Sequence (5ʹ-3ʹ)
CT-F3	GAACAGA(A/T)GC(T/A)GCGACAG
CT-B3	(C/T)GGGTTTAGAGTAGT(G/A)A(C/T)ATC
CT-FIP	TTAACTCCAATGTA(A/G)GGAGTGAACA-ATGCCTCTATTGA(C/T)TACCAT
CT-BIP	GGTCT(A/C)GAG(T/C)AAGTTTTGATGCCG–CAAGAT(T/A)GCTT(C/T)AGCCAATT
CT-LF	CATATT(T/C)A(AAT(C/T)CGTATAGCTCAGCC
CT-LB	AT(C/T)CGTATAGCTCAGCC
CT-AuNP	SH-AAAAAAAAAAGA(G/A)TGGCAAGCAAGTTTA

**Abbreviations:** AuNP, gold nanoparticle probe; B, reverse; CT, *Chlamydia trachomatis*; F, forward; IP, initial loop; L, loop.

### Optimization of LAMP-AuNP

To determine the optimal LAMP assay condition (incubation temperature and incubation time), the LAMP reaction was incubated at 59°C to 65°C for 30 to 60 minutes (without loop primers) and 10 to 35 minutes (with loop primers). A 25 μL LAMP reaction comprised DNA template (from High Pure PCR Template Preparation Kit, Roche Diagnostics), 1×buffer (20 mM Tris-HCl, 10 mM (NH_4_)_2_SO_4_, 10 mM KCl, 2 mM MgSO_4_, 0.1% Triton X-100 [v/v] [pH 8.8]), 1.6 μM primers CT-FIP and CT-BIP, 0.2 μM primers CT-F3 and CT-B3, 1.4 μM primers CT-LF and CT-LB, 1.4 mM dNTP (SibEnzyme Ltd., Novosibirsk, Russia), 0.5 M Betaine (Sigma-Aldrich, St. Louis, MO), 6 mM MgSO_4_, and 8 U *Bst* DNA polymerase (New England Biolabs, Beverly, MA). The protocols for DNA-functionalized AuNPs were as established [4,7]. Nanogold particles of 10 nm were appended to the probe (Table 1) to create the complementary AuNP-DNA probe specific for the *C*. *trachomatis* LAMP product. For colorimetric detection, the LAMP product and the DNA-functionalized AuNPs were hybridized at 61°C for 7 minutes, and then 0.01 M to 1 M of salt (NaCl or MgSO_4_) was added. The optimal LAMP condition, LAMP-to-AuNP ratio (vol/vol), and salt concentration for the LAMP-AuNP were each determined as the shortest assay time that yields the proper test specificity results and the lowest LOD. The result could be interpreted by the naked eye via a red (positive) or purple/blue/gray (negative) color change, and by UV-vis spectrophotometry. Controls included the AuNPs only, AuNPs with salt, and a no-template control: AuNPs only show the same color as the positive LAMP results, and the AuNPs with salt and the no-template control show the same color as the negative LAMP results. When adding an appropriate salt concentration, the AuNPs agglomerate and change color from red to purple/blue/gray; however, the positive LAMP-products hybridize the AuNPs via specific complementarity, thus inhibiting the salt-induced agglomeration [7]. All controls showed the correct colors for all tests.

### *C*. *trachomatis* detection by crude DNA lysis and LAMP-AuNP

The POC was evaluated with 130 endocervical swab samples for crude DNA lysis followed by LAMP-AuNP and LAMP-GE. These were compared to a commercial DNA extraction kit followed by PCR-GE. Crude DNA lysis was achieved by mixing 40 μl of the endocervical swab sample in M4RT (or Tris-EDTA buffer: 10 mM Tris-Cl and 1 mM EDTA [pH 7.5]) with 20 μl of Tris-EDTA buffer, then boiling at 95°C for 5 minutes using a heat block or water bath. The tube was placed on ice, and 5 μL was added to the 25 μL LAMP reaction comprising the aforementioned recipe. The optimized LAMP reaction conditions (61°C for 20 to 35 minutes), LAMP-to-AuNP volume ratio (5:5), and MgSO_4_ concentration (0.048 M) for the LAMP-AuNP hybridization were utilized. Assay accuracy was calculated based on the following equations: sensitivity = true positive ÷ (true positive + false negative), specificity = true negative ÷ (true negative + false positive), false positive = 1 − specificity, false negative = 1 − sensitivity, and accuracy = (true positive + true negative) ÷ total samples. Verification of all samples included a minimum of two independently confirmed commercial DNA extraction and PCR-GE results followed by *ompA* sequencing [2]. For negative results, the samples were additionally validated for the presence of human housekeeping gene via human *β-globin* PCR [2]. All negative results showed the human *β-globin* [2].

## Results

### Optimization of LAMP-AuNP

**Fig 1:**
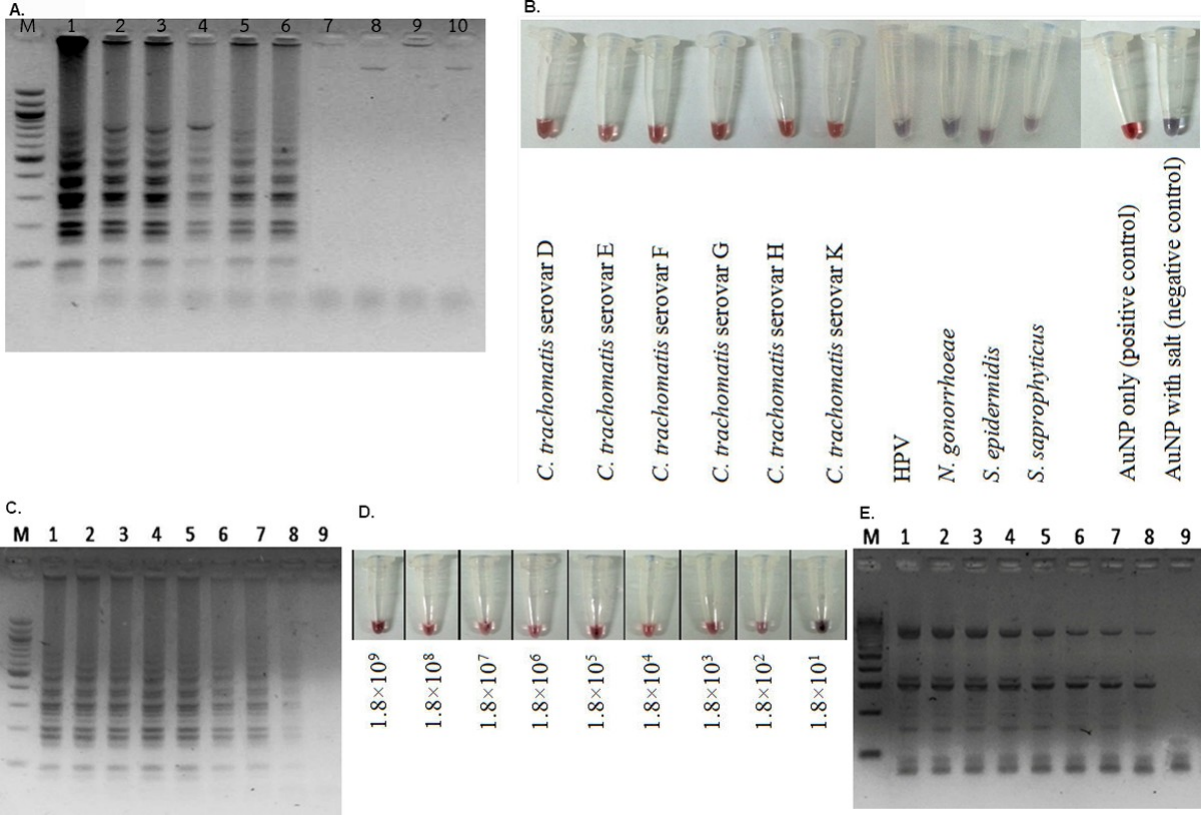
**Detection of *C*. *trachomatis* by LAMP-GE (A and C), LAMP-AuNP (B and D), and PCR-GE (E).** (A, B) Specificity determination using *C*. *trachomatis* serovars D, E, F, G, H, and K (from left to right, lane numbers 1–6) and HPV, *N*. *gonorrhoeae*, *S*. *epidermidis*, and *S*. *saprophyticus* (lanes 7–10). Additional controls in B included AuNPs only and AuNPs with salt. (C, D, E) The positive results were observed from lanes 1 to 8, representing a detection limit of 180 genome copy numbers of *ompA* (equivalent to 0.085 fg of *ompA*), using *C*. *trachomatis* strain D. Lane 9 is 18 genome copy numbers of *ompA* (equivalent to 0.017 fg of *ompA*). M represents GeneRuler 100 bp plus DNA ladder (Invitrogen, New York). AuNP, gold nanoparticle probe; *Ct*, *Chlamydia trachomatis*; GE, gel-electrophoresis; HPV, human papillomavirus; LAMP, loop-mediated isothermal amplification; LOD, limit of detection; *ompA*, outer membrane protein A; PCR, polymerase chain reaction.

**Fig 2:**
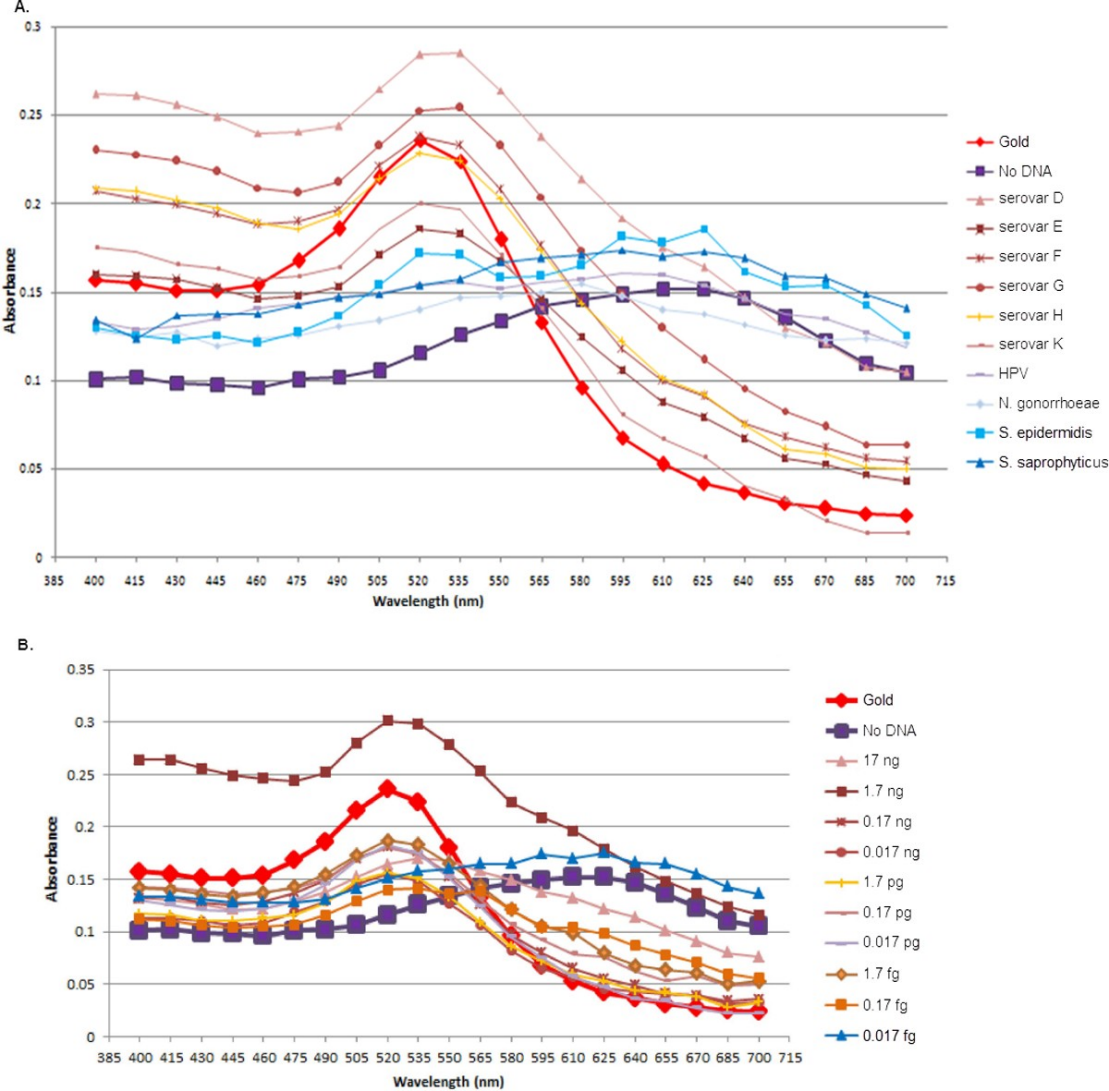
**LAMP-AuNP followed by UV-vis spectrophotometry of the corresponding specificity (A) and sensitivity (B) assays in Fig 1B and 1D, respectively.** The x-axis represents wavelength (nm). A shift of peak to a higher wavelength was observed for the no-template and negative test results. AuNP, gold nanoparticle probe; HPV, human papillomavirus; LAMP, loop-mediated isothermal amplification.

Loop primers LF and LB, which specifically locate the dumbbell products of LAMP, allow for extra amplification of the loop amplicons. Therefore, a sufficient amount of amplicons for detection by GE (or AuNP) were made at the shorter assay time. S1 Fig shows that the loop primers can speed up the LAMP reaction time from 30 to 17.5 minutes at 61°C [8]. For the hybridization condition with the AuNP, the optimized ratio of LAMP-to-AuNP volume of 5:5 (S2 Fig), a salt (MgSO_4_) concentration of 0.048 M, and an adjustment of the LAMP reaction time to 20 minutes were all required to yield the most vivid color of the LAMP-AuNP hybridization product. The specificity of the LAMP-AuNP was compared along the LAMP-GE among the prevalent STD *C*. *trachomatis* serovars (D to K), other STD pathogens (human papillomavirus [HPV], *N*. *gonorrhoeae*, and *Staphylococcus saprophyticus*), and a typical skin bacteria like *S*. *epidermidis* (Fig 1A and 1B). A common fungal STD pathogen *Candida albicans* was also included to confirm there was no cross-reaction to fungi (S3 Fig). All results showed the positive reaction specific to only *C*. *trachomatis*. The color visualization of the LAMP-AuNP product was confirmed by UV-vis spectrophotometry (Fig 2A) [4,7].

For determination of the LOD of the assay, 10-fold serial dilutions (1.8×10^9^ to 1.8×10^1^ *C*. *trachomatis* genome equivalents) were prepared. The positive results are shown, starting at 1.8×10^2^ or 180 copies for LAMP-GE (Fig 1C), LAMP-AuNP (Fig 1D), and PCR-GE (Fig 1E). Following this, 2-fold serial dilutions from 180 to 90, 45, 22.5, and 11.25 copies (0.085 fg, 0.0425 fg, 0.02125 fg, and 0.010625 fg) were performed to further determine the LOD. The results demonstrated an LOD of 22.5 copies for PCR-GE and 45 copies for LAMP-GE and LAMP-AuNP. Moreover, increasing the LAMP reaction incubation from 20 to 35 minutes allowed the LOD to become consistent at 11.25 copies (S4 Fig, lane 1). The colorimetric change of the LAMP-AuNP was confirmed by UV-vis spectrophotometry [7]. Therefore, the LAMP-AuNP has an LOD of 45 copies when incubated for 20 minutes and 11.25 copies when incubated for 35 minutes. The color of the LAMP-AuNP samples could be detected by the naked eye, similar to Fig 1B and 1D. This LOD was comparable to the LAMP-GE and PCR-GE and to that of the published microfluidics PCR methods for POC *C*. *trachomatis* diagnosis [9].

### Evaluation using real clinical samples

The testing of feasibility and accuracy for POC diagnostics included a 5-minute crude DNA lysis to LAMP-AuNP assay based on 96 symptomatic and 34 healthy endocervical swab samples from women in Bangkok and rural provinces. The assay was run in a blinded fashion and compared to PCR-GE and LAMP-GE. For the clinical sample collection medium for POC (immediate) diagnosis, we found that M4RT is not required: swabs can be collected in, for example, Tris-EDTA buffer. Of the 130 clinical samples, the LAMP-AuNP sensitivity was 96% (23/24) and specificity 99% (105/106). This result is higher than the LAMP-GE (Table 2). The percentages of assay accuracy for PCR-GE and LAMP-AuNP were equal (98%), whereas for LAMP-GE it was 96%. The AuNPs allow simple visualization of results and improve the LAMP specificity and sensitivity [7]. Independent replicates and comparisons were performed to support the statistical findings of the assay effectiveness (sensitivity, specificity, false positive, and false negative rates) (S1 Table). The percentages of assay accuracy for the independent replicate detection were 100% for PCR-GE, 99% for LAMP-AuNP, and 98% for LAMP-GE. Verification from the UV-vis spectra supported the convenient colorimetric reading of the LAMP-AuNP assay [7]. The A535/A595 ratio of >1.00 was found to correlate with positive results (24/25) and <1.00 with negative results (106/105). Furthermore, an approximately 10-fold less amount of the endocervical swab sample is required for this POC: 5 μl elution is equal to 3 μl of original sample for LAMP, versus 1 μl to 3 μl elution, which equates to 10 μl to 30 μl original sample for PCR. Together, the data demonstrated the reliability of our *C*. *trachomatis* POC diagnostic by crude DNA lysis and LAMP-AuNP in clinical endocervical swab samples. This eliminates the requirement of DNA extraction and purification, which adds cost and time to diagnosis [3]. Our approach also decreases the amount of clinical sample required from 10–30 μl down to 3 μl of original sample, all without a thermal cycler, electrophoresis, or spectrophotometry. A simple heat block or water bath along with the reagents are sufficient to perform the *C*. *trachomatis* LAMP-AuNP detection at high sensitivity and specificity at the POC, which is appropriate for local and resource-constrained settings.

**Table 2:** Clinical sample evaluation of crude DNA lysis combined LAMP-GE and LAMP-AuNP, compared with a commercial DNA extraction kit and PCR-GE. For specificity, the values in parentheses represent the number of true negatives divided by the number of true negatives and false positives. For sensitivity, the values in parentheses represent the number of true positives divided by the number of true positives and false negatives.

Clinical Groups (number of samples)	Test Statistics	PCR-GE	LAMP-GE	LAMP-AuNP
Symptomatic STD (96)	Specificity	0.97 (70/72)	0.96 (69/72)	0.99 (72/73)
	Sensitivity	1.00 (24/24)	0.92 (22/24)	0.96 (22/23)
	False positive	0.03	0.04	0.01
	False negative	0.00	0.08	0.04
Healthy (34)	Specificity	1.00 (33/33)	1.00 (33/33)	1.00 (33/33)
	Sensitivity	1.00 (1/1)	1.00 (1/1)	1.00 (1/1)
	False positive	0.00	0.00	0.00
	False negative	0.00	0.00	0.00
Total (130)	Specificity	0.98 (103/105)	0.97 (102/105)	0.99 (105/106)
	Sensitivity	1.00 (25/25)	0.92 (23/25)	0.96 (23/24)
	False positive	0.02	0.03	0.01
	False negative	0.00	0.08	0.04
Detection limit (copies)		22.5	11.25–45	11.25–45*
Diagnosis time (h)		4:46	1:02–1:17	0:32–0:47*
USD Price/reaction^†^		30	3	3

* The detection limit of LAMP reduced from 45 to 11.25 copies when the LAMP reaction was incubated at 61°C for 35 minutes (45 copies when the reaction was incubated at 61°C for 20 minutes). Therefore, diagnosis time and detection limit of LAMP have two numbers, depending on the reaction time.

†The differences in the nucleic acid preparation step between PCR (using High Pure Template Preparation Kit, Roche Diagnostics, Indianapolis, IN) and LAMP (heat-treated) were excluded in price per reaction. The price per reaction of the High Pure Template Preparation Kit is approximately 3 USD.

**Abbreviations:** AuNP, gold nanoparticle probe; GE, gel-electrophoresis; LAMP, loop-mediated isothermal amplification; LOD, limit of detection; PCR, polymerase chain reaction; USD, United States dollars.

## Discussion

The *C*. *trachomatis* LAMP-AuNP detection (pending petty patent no. 1503002153) is appropriate for local and resource-constrained settings. The assay directly uses clinical samples, obtains results in 32 to 47 minutes (5 minutes crude DNA lysis, 20 to 35 minutes LAMP depending on the preferred LOD, and 7 minutes AuNP hybridization), and the result is readable by the naked eye. Clinical evaluation showed that the LOD, specificity (99% to100%), and sensitivity (96%) are all comparable with PCR-GE [1,10]. Additionally, urine samples may be tested to allow this POC diagnostic in males. Studies on a larger sample size and a commercial (FDA-approved) kit comparison are ongoing to further validate this assay as a screening method for sexually-transmitted *C*. *trachomatis* infections.

## Supporting information

S1 Checklist STARD checklist. STARD, Standards of Reporting of Diagnostic Accuracy.
(DOCX)

S1 Flow diagram Prototypical STARD diagram to report flow of participants through the study. STARD, Standards of Reporting of Diagnostic Accuracy.
(PDF)

S1 Fig **LAMP-GE without (A) and with (B) inner loop primers.** (A) Different incubation temperatures (59°C to 65°C) and incubation periods (30 to 60 minutes) were tested. An optimal temperature was found at 61°C. (B) Shorter incubation periods (lanes 1–6: 10 minutes, 12.5 minutes, 15 minutes, 17.5 minutes, 20 minutes, and 22.5 minutes, respectively) were tested after the inner loop primers were added. M represents GeneRuler 100 bp plus DNA ladder. DNA template contained 100 ng of purified C. trachomatis strain D DNA using High Pure PCR Template Preparation Kit (Roche Diagnostics). GE, gel-electrophoresis; LAMP, loop-mediated isothermal amplification; PCR, polymerase chain reaction.
(TIF)

S2 Fig Optimized ratios of LAMP-to-AuNP volume for optimal color contrast between positive and negative test results. Positive, red; Negative, purple/blue/gray. AuNP, gold nanoparticle probe; LAMP, loop-mediated isothermal amplification.
(TIF)

S3 Fig Specificity determination of LAMP-GE against fungal *Candida albicans*. The first lane is GeneRuler 100 bp plus DNA ladder, followed by a no template control, and *C*. *albicans* lanes, respectively. GE, gel-electrophoresis; LAMP, loop-mediated isothermal amplification.
(TIF)

S4 Fig LOD of *C*. *trachomatis omp*A at 11.25 and 22.5 genome equivalent copies (0.010625 fg and 0.02125 fg), lanes 1 and 2, respectively, by LAMP-GE. M represents 100 bp DNA Ladder RTU (GeneDireX, Inc., Miaoli County, Taiwan). The LAMP reaction was as described in the legend of Fig 1, with incubation at 61°C for 35 minutes. GE, gel-electrophoresis; LAMP, loop-mediated isothermal amplification; *ompA*, outer membrane protein A.
(TIF)

S1 Table Clinical sample evaluation of crude DNA lysis combined LAMP-GE and LAMP-AuNP, compared with a commercial DNA extraction kit and PCR-GE, for the independently replicated assays. For specificity, the values in parentheses represent the number of true negatives divided by the number of true negatives and false positives. For sensitivity, the values in parentheses represent the number of true positives divided by the number of true positives and false negatives. AuNP, gold nanoparticle probe; GE, gel-electrophoresis; LAMP, loop-mediated isothermal amplification; PCR, polymerase chain reaction.
(DOCX)
