# Supplementary figures and images for: Rapid and sensitive detection of Chlamydia trachomatis sexually transmitted infections in resource-constrained settings in Thailand at the point-of-care
Source: PLoS Negl Trop Dis. 2018 Dec 20;12(12):e0006900. doi: 10.1371/journal.pntd.0006900 (PMC6301561; doi:10.1371/journal.pntd.0006900)

# Prototypical STARD diagram to report flow of participants through the study

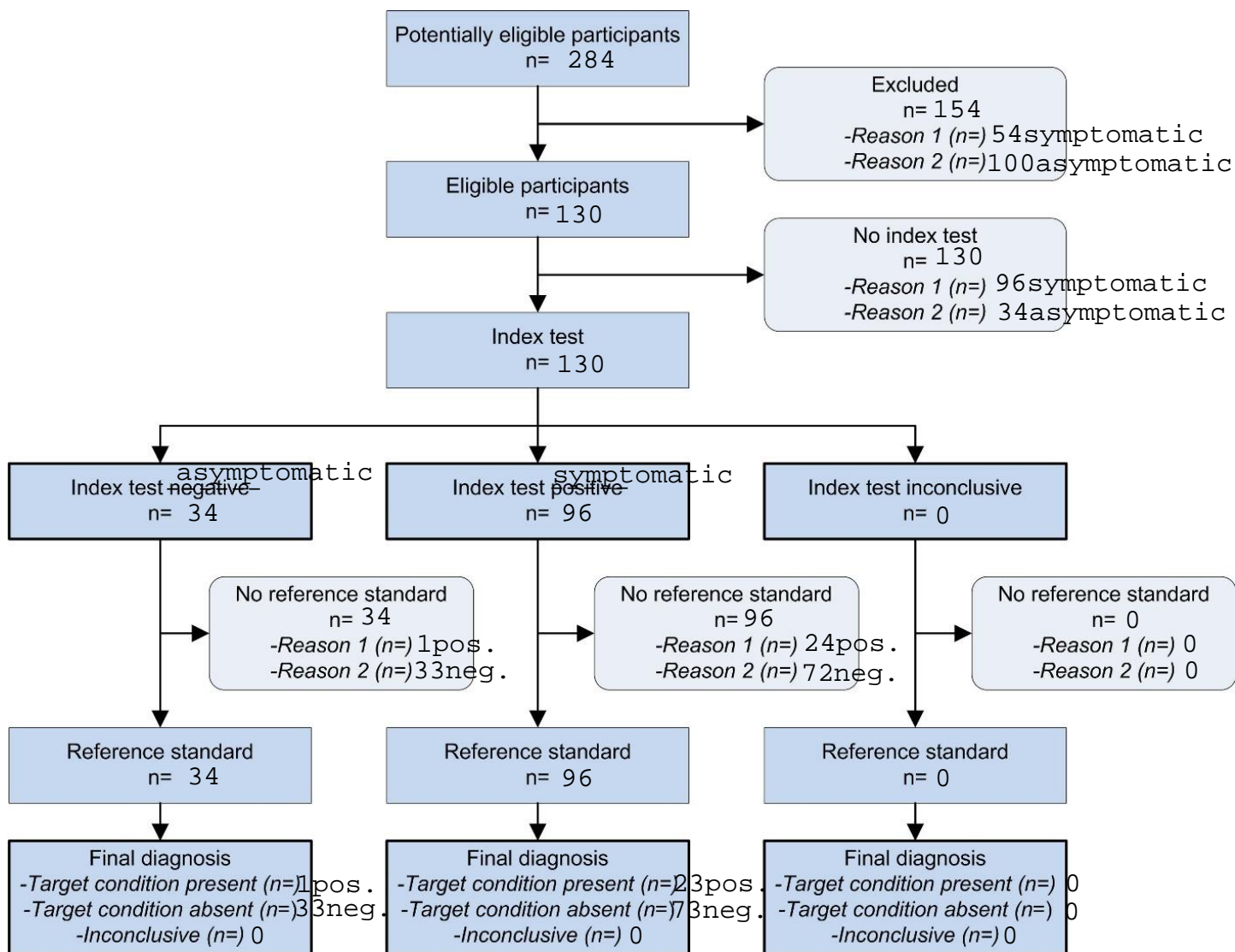

Supplement: S1 Flow diagram — STARD, Standards of Reporting of Diagnostic Accuracy. (PDF) [file pntd.0006900.s002.pdf]

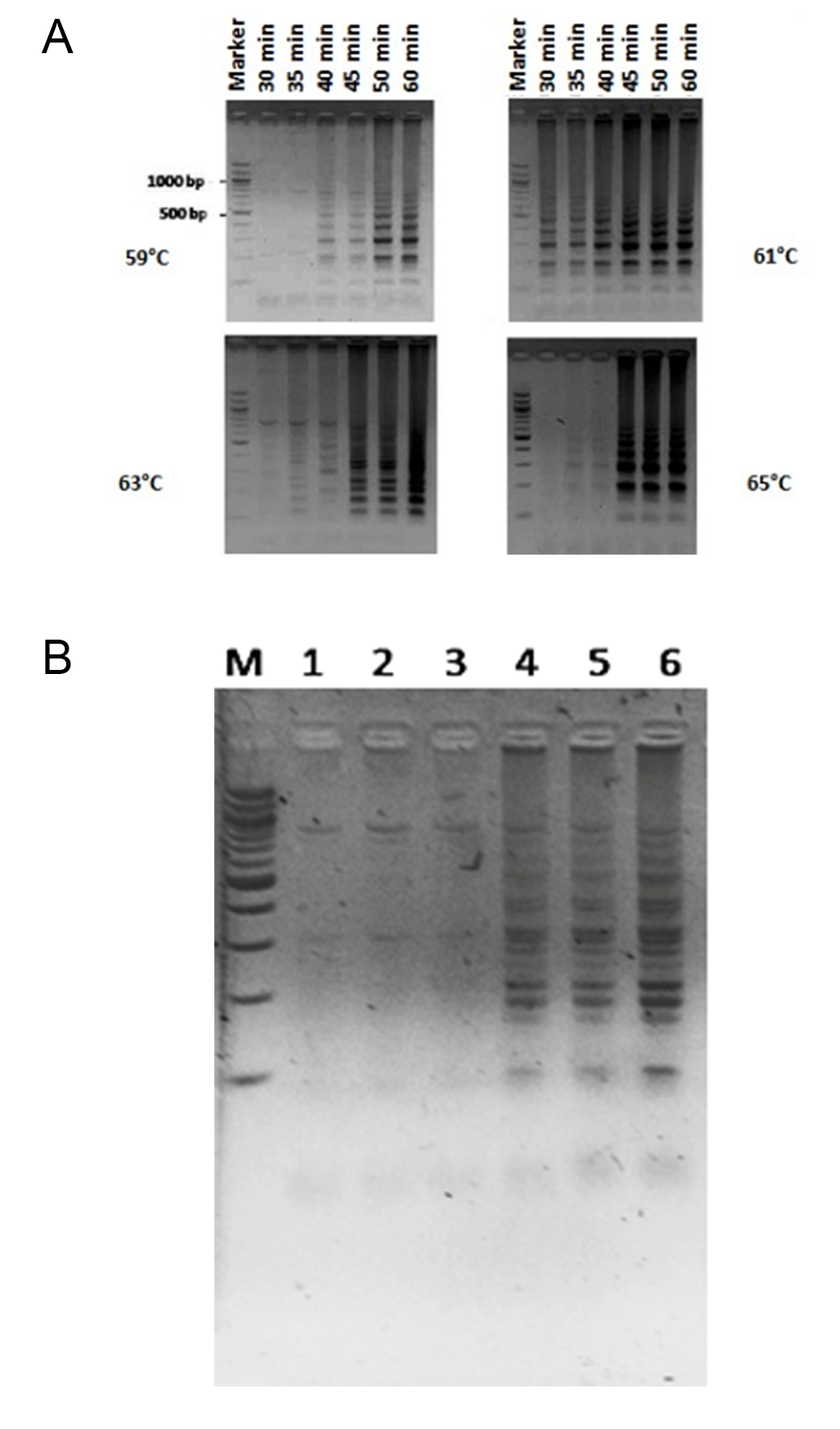

Supplement: S1 Fig — LAMP-GE without (A) and with (B) inner loop primers. (A) Different incubation temperatures (59°C to 65°C) and incubation periods (30 to 60 minutes) were tested. An optimal temperature was found at 61°C. (B) Shorter incubation periods (lanes 1–6: 10 minutes, 12.5 minutes, 15 minutes, 17.5 minutes, 20 minutes, and 22.5 minutes, respectively) were tested after the inner loop primers were added. M represents GeneRuler 100 bp plus DNA ladder. DNA template contained 100 ng of purified C. trachomatis strain D DNA using High Pure PCR Template Preparation Kit (Roche Diagnostics). GE, gel-electrophoresis; LAMP, loop-mediated isothermal amplification; PCR, polymerase chain reaction. (TIF) [file pntd.0006900.s003.tif]

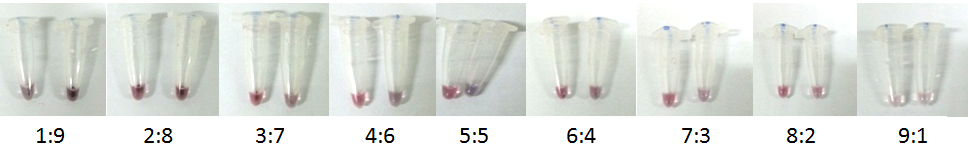

Supplement: S2 Fig — Positive, red; Negative, purple/blue/gray. AuNP, gold nanoparticle probe; LAMP, loop-mediated isothermal amplification. (TIF) [file pntd.0006900.s004.tif]

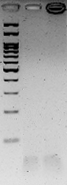

Supplement: S3 Fig — The first lane is GeneRuler 100 bp plus DNA ladder, followed by a no template control, and C. albicans lanes, respectively. GE, gel-electrophoresis; LAMP, loop-mediated isothermal amplification. (TIF) [file pntd.0006900.s005.tif]

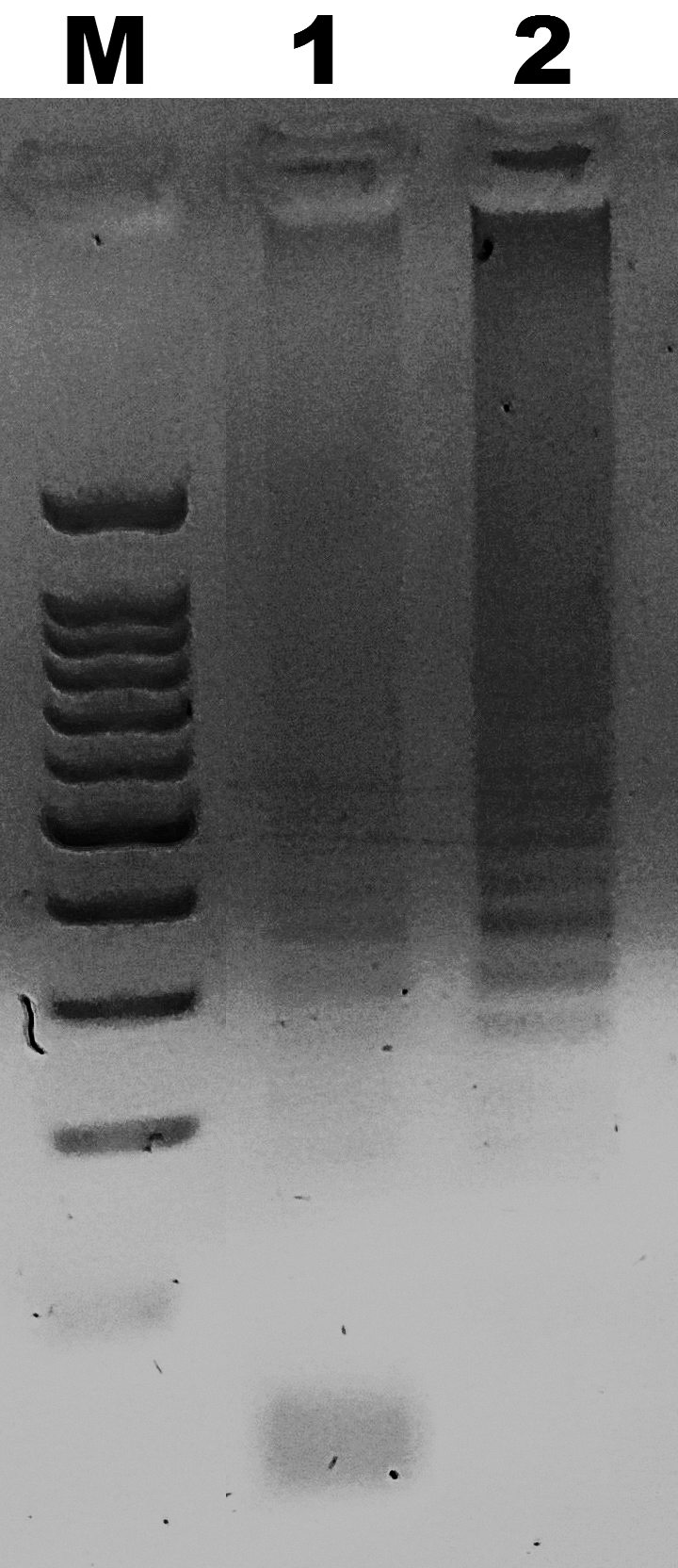

Supplement: S4 Fig — M represents 100 bp DNA Ladder RTU (GeneDireX, Inc., Miaoli County, Taiwan). The LAMP reaction was as described in the legend of Fig 1, with incubation at 61°C for 35 minutes. GE, gel-electrophoresis; LAMP, loop-mediated isothermal amplification; ompA, outer membrane protein A. (TIF) [file pntd.0006900.s006.tif]
