# Supplementary material for: Rapid and sensitive detection of Chlamydia trachomatis sexually transmitted infections in resource-constrained settings in Thailand at the point-of-care
Source: PLoS Negl Trop Dis. 2018 Dec 20;12(12):e0006900. doi: 10.1371/journal.pntd.0006900 (PMC6301561; doi:10.1371/journal.pntd.0006900)
Supplement: S1 Table — For specificity, the values in parentheses represent the number of true negatives divided by the number of true negatives and false positives. For sensitivity, the values in parentheses represent the number of true positives divided by the number of true positives and false negatives. AuNP, gold nanoparticle probe; GE, gel-electrophoresis; LAMP, loop-mediated isothermal amplification; PCR, polymerase chain reaction. (DOCX) [file pntd.0006900.s007.docx]

**S1 Table.**

| **Clinical Groups (no. of samples)** | **Test Statistics** | **PCR-GE** | **LAMP-GE** | **LAMP-AuNP** |
| --- | --- | --- | --- | --- |
| Symptomatic STD (96) | Specificity | 1.00 (72/72) | 0.99 (71/72) | 1.00 (72/72) |
|  | Sensitivity | 1.00 (24/24) | 0.96 (23/24) | 0.96 (23/24) |
|  | False Positive | 0.00 | 0.01 | 0.00 |
|  | False Negative | 0.00 | 0.04 | 0.04 |
| Healthy (34) | Specificity | 1.00 (33/33) | 1.00 (33/33) | 1.00 (33/33) |
|  | Sensitivity | 1.00 (1/1) | 1.00 (1/1) | 1.00 (1/1) |
|  | False Positive | 0.00 | 0.00 | 0.00 |
|  | False Negative | 0.00 | 0.00 | 0.00 |
| Total (130) | Specificity | 1.00 (105/105) | 0.99 (104/105) | 1.00 (105/105) |
|  | Sensitivity | 1.00 (25/25) | 0.96 (24/25) | 0.96 (24/25) |
|  | False Positive | 0.00 | 0.01 | 0.00 |
|  | False Negative | 0.00 | 0.04 | 0.04 |
